# Sex Differences in Children and Adolescents With Hypertrophic Cardiomyopathy

**DOI:** 10.1016/j.jacadv.2025.101907

**Published:** 2025-07-05

**Authors:** Gabrielle Norrish, Kimberley Hall, Ella Field, Elena Cervi, Olga Boleti, Lidia Ziółkowska, Iacopo Olivotto, Sylvia Passantino, Diala Khraiche, Giuseppe Limongelli, Robert G. Weintraub, Aris Anastasakis, Elena Biagini, Luca Ragni, Georgia Sarquella-Brugada, Sergi Cesar, Terrence Prendiville, Karen McLeod, Maria Ilina, Anwar Baban, Tiina H. Ojala, Georgia Spentzou, Vinay Bhole, Feran Gran, Elspeth Brown, Grazia Delle Donne, Bernadette Khodaghalian, Adrian Fernandez, Piers E.F. Daubeney, Katie Linter, Peter Kubus, Orhan Uzun, Regina Bökenkamp, Francesca Raimondi, Chiara Marrone, Constantio Medrano, Esther Gonzalez-Lopez, Ana Siles, Katarzyna Luczak-Wozniak, Tara Bharucha, Satish Adwani, Sabine Klaassen, Fernando J. Castro, Luis Guereta, Hirokuni Yamazawa, Gianfranco Sinagra, Anca Popoiu, Francesca Perin, B. Chana, Hans De Wilde, Torsten.B. Rasmussen, Jens Mogensen, Sujeev Mathur, Fernando Centeno, Zdenka Reinhardt, Roberto Barriales-Villa, Toru Kubo, Tiziana Felice, Cristina Radulescu, Sylvie Schouvey, Melissa Chaker, Juan Pablo Kaski

**Affiliations:** a Centre for Inherited Cardiovascular Diseases, Great Ormond Street Hospital, London, United Kingdom; b Department of Cardiology, Institute of Cardiovascular Sciences University College London, London, United Kingdom; c Department of Cardiology, University of Exeter, Exeter, United Kingdom; d Department of Cardiology, The Children’s Memorial Health Institute, Warsaw, Poland; e Department of Cardiology, Meyer Children’s Hospital IRCSS, Florence, Italy; f Department of Cardiology, Necker –Enfants Malades Hospital, Paris, France; g Department of Cardiology, Monaldi Hospital, Naples, Italy; h Department of Cardiology, The Royal Children’s Hospital, Melbourne, Australia; i Department of Cardiology, University of Melbourne, Melbourne, Australia; j Department of Cardiology, Murdoch Children’s Research Institute, Parkville, Australia; k Department of Cardiology, Onassis Cardiac Surgery Center, Athens, Greece; l Cardiology Unit, Cardiac Thoracic and Vascular Department, IRCCS Azienda Ospedaliero-Universitaria di Bologna, Bologna, Italy; m European Reference Network for Rare, Low Prevalence and Complex Diseases of the Heart-ERN GUARD-Heart, Amsterdam, the Netherlands; n Arrhythmia, Inherited Cardiac Diseases and Sudden Death Unit, Hospital Sant Joan de Déu, Esplugues de Llobregat, Barcelona, Spain; o Arrítmies Pediàtriques, Cardiologia Genètica i Mort Sobtada, Malalties Cardiovasculars en el Desenvolupament, Institut de Recerca Sant Joan de Déu (IRSJD), Esplugues de Llobregat, Barcelona, Spain; p Medical Science Department, School of Medicine, Universitat de Girona, Girona, Spain; q Pediatrics Department, School of Medicine, Universitat de Barcelona, Barcelona, Spain; r Department of Cardiology, Our Lady’s Children’s Hospital, Dublin, Ireland; s Department of Cardiology, Royal Hospital for Children, Glasgow, United Kingdom; t Department of Cardiology, Bambino Gesu Hospital, Rome, Italy; u Department Clinical Molecular Genetics and Precision Medicine, Mediclinic City Hospital, Dubai, UAE; v Department of Pediatric Cardiology, New Children’s Hospital, University of Helsinki, Helsinki, Finland; w Department of Cardiology, Bristol Royal Hospital for Children, Bristol, United Kingdom; x Department of Cardiology, Birmingham Children’s Hospital, Birmingham, United Kingdom; y Department of Cardiology, Val D’Hebron University Hospital, Barcelona, Spain; z Department of Cardiology, Leeds General Infirmary, Leeds, United Kingdom; aa Department of Cardiology, Alder Hey Children’s Hospital, Liverpool, United Kingdom; ab Department of Cardiology, Fundación Favaloro University Hospital, Buenos Aires, Argentina; ac Department of Cardiology, Royal Brompton and Harefield NHS Trust, London, United Kingdom; ad Department of Cardiology, Glenfield Hospital, Leicester, United Kingdom; ae Department of Cardiology, University Hospital Motol, Prague, Czech Republic; af Department of Cardiology, University Hospital of Wales, Cardiff, United Kingdom; ag Department of Cardiology, Leiden University Medical Center, Leiden, the Netherlands; ah Department of Cardiology, Fondazione Toscana G Monasterio, Massa-Pisa, Pisa, Italy; ai Department of Cardiology, Papa Giovanni XXIII Hospital, Bergamo, Italy; aj Department of Cardiology, Hospital General Universitario Gregorio Marañón, Madrid, Spain; ak Heart Failure and Inherited Cardiac Diseases Unit, Department of Cardiology, Hospital Universitario Puerta de Hierro Majadahonda, IDIPHISA, CIBERCV, Madrid, Spain; al Department of Pediatrics, Hospital Universitario Puerta de Hierro Majadahonda, Madrid, Spain; am Department of Pediatric Cardiology and General Pediatrics, Medical University of Warsaw, Warsaw, Poland; an Department of Cardiology, Southampton General Hospital, Southampton, United Kingdom; ao Department of Cardiology, John Radcliffe Hospital, Oxford, United Kingdom; ap Department of Pediatric Cardiology, Deutsches Herzzentrum der Charité, Berlin, Germany; aq Experimental and Clinical Research Center (ECRC), a Joint Cooperation Between the Charité Medical Faculty and the Max-Delbrück-Center for Molecular Medicine (MDC), Charite – Universitatsmedizin Berlin, Berlin, Germany; ar DZHK (German Centre for Cardiovascular Research), Partner Site Berlin, Berlin, Germany; as Department of Cardiology, University Hospital Virgen de la Arrixaca, Murcia, Spain; at Department of Cardiology, University Hospital La Paz, Madrid, Spain; au Department of Pediatrics, Faculty of Medicine and Graduate School of Medicine, Hokkaido University Hospital, Sapporo, Japan; av Heart Muscle Disease Registry Trieste, University of Trieste, Trieste, Italy; aw University of Medicine and Pharmacy "Victor Babes" Timisoara, Department of Pediatrics, Children’s Hospital ‘Louis Turcanu’, Timisoara, Romania; ax Department of Cardiology, Hospital Universitario Virgen de las Nieves, Granada, Spain; ay Department of Cardiology, Ospedaliero Universitaria di Parma, Parma, Italy; az Department of Cardiology, Ghent University Hospital, Ghent, Belgium; ba Department of Cardiology, Aarhus University Hospital, Aarhus, Denmark; bb Department of Cardiology, Odense University Hospital, Odense, Denmark; bc Department of Cardiology, Evelina Children’s Hospital, London, United Kingdom; bd Department of Cardiology, Rio Hortega University Hospital, Valladolid, Spain; be Department of Cardiology, The Freeman Hospital, Newcastle, United Kingdom; bf Complexo Hospitalario Universitario A Coruna, INIBIC, CIBERCV, Coruna, Spain; bg Department of Cardiology, Kochi Medical School Hospital, Kochi, Japan; bh Department of Cardiology, Mater Dei Hospital, Msida, Malta; bi Department of Cardiology, UMPCD Bucharest, Bucharest, Romania; bj Department of Cardiology, Hospital Saint Joseph, Marseille, France; bk Department of Cardiology, Hospital Garrahan, Buenos Aires, Argentina

**Keywords:** adolescent, hypertrophic cardiomyopathy, phenotype, sex differences

## Abstract

**Background:**

Sex differences have been described in adults with hypertrophic cardiomyopathy (HCM), but it is unknown if similar differences exist in childhood-onset disease.

**Objectives:**

This study aimed to investigate the influence of biological sex on the clinical characteristics and outcomes of children with HCM.

**Methods:**

An international retrospective cohort of patients diagnosed with nonsyndromic HCM ≤16 years was formed. Sex differences in baseline characteristics and clinical outcomes were investigated. Primary outcome was all-cause mortality or cardiac transplantation. Secondary outcomes include major arrhythmic cardiac event and heart failure event.

**Results:**

Of 1,433 patients diagnosed at a median age of 11 years (IQR: 6-14), 471 (33.0%) were female. Although there were no sex differences in phenotype in preadolescent patients (<12 years), adolescent female patients were more likely to have heart failure symptoms (n = 53 [31.9%] vs n = 86 [22.5%]; *P* = 0.019). Adolescent female patients had larger left atrial size (1.4 *z*-score [±2.3] vs 2.1 *z*-score [±2.5]; *P* = 0.0056) but there was no difference in degree of hypertrophy or proportion with obstructive disease. Over a median follow-up of 5.3 years (IQR: 2.9, 8.0) annual incidence of all-cause mortality or cardiac transplantation, major arrhythmic cardiac event or heart failure events did not vary by sex.

**Conclusions:**

Young female patients with HCM are more likely to experience heart failure symptoms and have echocardiographic features of diastolic impairment. Despite differences in phenotype, outcomes during childhood and young adulthood are not different. Further studies are required to explore the underlying mechanisms for these observed differences.

Hypertrophic cardiomyopathy (HCM) is a heterogeneous disease of the heart muscle, most commonly caused by sarcomere protein gene variants and characterized by age-related incomplete penetrance and variable long-term outcomes. In common with other cardiovascular (CV) diseases, important sex differences have been described in adults with HCM.^1^ Women are typically older at the time of diagnosis, more likely to have heart failure symptoms at presentation, and have a higher prevalence of left ventricular outflow tract obstruction (LVOTO) and diastolic impairment.^2^, ^3^, ^4^, ^5^, ^6^, ^7^, ^8^ Despite the autosomal dominant pattern of inheritance of sarcomeric variants, men are overrepresented in published HCM cohorts, accounting for approximately 60%,^2^, ^3^, ^4^, ^5^, ^6^, ^7^ but women are more likely to have a sarcomeric variant identified on genetic testing.^9^ Furthermore, outcomes also differ between the sexes, with disease-related excess mortality higher in women, largely secondary to heart failure-related deaths.^2^^,^^6^^,^^10^ The mechanisms underlying these observed sex differences remain poorly understood and are likely multifactorial, including biological (eg, sex hormone effect^11^) and nonbiological (eg, societal and cultural effects^12^) factors.

Childhood-onset HCM has a similar genetic basis to adult-onset disease^13^ but has a distinct natural history, with a higher prevalence of arrhythmic events and need for cardiac transplantation.^14^^,^^15^ It is unknown whether similar sex differences to those previously described in adults exist in childhood cohorts. The aim of this study was to describe sex differences in the presentation, phenotype, and outcomes of children with early-onset HCM in a large international cohort.

## Methods

### Study population

Children diagnosed with HCM aged 0-≤16 years of age were identified from the International Paediatric Hypertrophic Cardiomyopathy Consortium. HCM was defined as a maximal left ventricular wall thickness (MLVWT) >2 SDs above body surface area (BSA)-corrected population mean (*z*-score ≥+2)^16^^,^^17^. Patients with a diagnosis of an inborn error of metabolism, RASopathy syndrome, or neuromuscular disease were excluded from this study.

### Data collection

Anonymized, noninvasive clinical data were collected from baseline evaluation and follow-up, including heart failure symptoms (NYHA or Ross functional classification for those younger than 5 years old^18^), family history, resting and ambulatory electrocardiography, transthoracic echocardiography (2-dimensional, Doppler, and color), and interventions (left ventricular myectomy, implantable cardiac defibrillator [ICD] implantation). The presence of heart failure symptoms was defined as a NYHA or Ross functional class ≥2. MLVWT and left atrial (LA) diameter measurements, obtained as previously described, are expressed in millimeters and *z*-scores relative to the distribution of measurements for BSA in healthy children.^17^ LVOT gradient was measured at rest. LVOTO was defined as an instantaneous peak Doppler LVOT pressure gradient ≥30 mm Hg. Genetic testing was performed at the discretion of the treating clinician as part of usual care. Patients were defined as having a “sarcomeric variant” if a sarcomeric pathogenic or likely pathogenic variant was identified on genetic testing.^19^ Data were collected independently at each participating center and data integrity is guaranteed by each author.

### Outcomes

The primary study outcome was all-cause mortality or cardiac transplantation occurring in childhood or young adulthood (defined a priori as age ≤21 years). Secondary outcomes were major arrhythmic cardiac event (MACE), defined as sudden cardiac death or an equivalent event (resuscitated cardiac arrest, appropriate ICD therapy for a ventricular tachyarrhythmia, or sustained ventricular tachycardia associated with hemodynamic compromise^20^) or heart failure event, defined as heart failure death or cardiac transplantation occurring in childhood or young adulthood. Outcomes were determined by the treating cardiologist at each center.

### Statistical analysis

The proportion of missing data is indicated for each data variable. Continuous variables are described as mean ± SD or median (IQR) as appropriate, with 3 group comparisons conducted using analysis of variance or Wilcoxon rank sum, respectively. Categorical variables were compared using the chi-square test. Follow-up was censored at the age of 21 years and 364 days as the aim of this study was to investigate the natural history of disease during childhood or young adulthood. Follow-up time was calculated from the date of their first evaluation in a participating center to the date of their most recent evaluation prior to the end of the study (August 2024) or until participants reached the age of 21 years and 364 days. The overall clinical characteristics of the cohort were compared between female and male patients and estimates of survival by sex were obtained using the Kaplan-Meier product limit method. Incidence rates were formed from the number of events divided by the person-time. CIs for incidence rates were calculated using the quadratic approximation to the Poisson log likelihood for the log-rate parameter. A log-rank test was used to compare survival distributions between the 2 groups. Statistical analysis was performed using Stata statistical software (StataCorp LLC) (Version 15).

### Ethics

This study conforms to the principles of the Helsinki Declaration and Good Clinical Practice. Local ethical approval was given for each participating center with a waiver of informed consent for retrospective, anonymized data.

## Results

**Figure 1:**
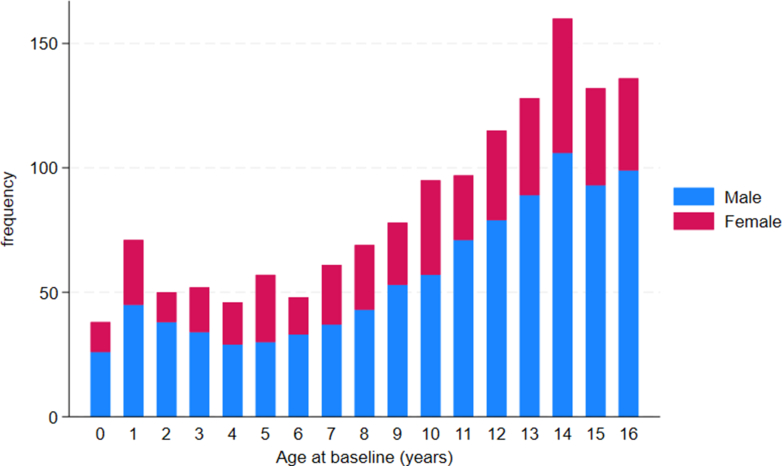
Sex Distribution by Age of Baseline Evaluation

Of 1,433 children diagnosed with nonsyndromic HCM at a median age of 11 years (IQR: 6-14), 962 (67%) were male. The age at baseline did not significantly differ by sex (Table 1) and a male predominance was seen throughout childhood (Figure 1, Central Illustration).

**Table 1:** Baseline Clinical Characteristics by Sex and Age

	Whole Cohort				Preadolescent (≤12 Years)				Adolescent (>12 Years)			
	Whole Cohort (N = 1,433)	Male (n = 962)	Female (n = 471)	*P* Value	Whole Cohort (N = 877)	Male (n = 575)	Female (n = 302)	*P* Value	Whole Cohort (N = 556)	Male (n = 387)	Female (n = 169)	*P* Value
Age at baseline, y	11 (6, 14)	11 (7, 14)	10 (6, 14)	0.0614	8 (4,10)	8 (4, 11)	7.5 (4, 10)	0.490	14 (14,15)	14 (14, 16)	14 (14,15)	0.409
Age groups												
Infantile	38 (2.7%)	26 (2.7%)	12 (2.6%)	0.364								
1-5 y	276 (19.3%)	176 (18.3%)	100 (21.2%)									
6-12 y	563 (39.3%)	373 (38.8%)	19 (40.3%)									
12+	556 (38.8%)	387 (38.8%)	169 (35.9%)									
Previous VF/VT (n = 1,416)	26 (1.8%)	14 (1.5%)	12 (2.6%)	0.346	17 (1.9%)	11 (1.9%)	6 (2.0%)	0.738	9 (1.6%)	3 (0.8%)	6 (3.6%)	0.020
Family history HCM (n = 1,401)	758 (53.4%)	504 (52.9%)	254 (54.4%)	0.866	458 (52.8%)	303 (53.3%)	155 (51.8%)	0.763	300 (54.4%)	201 (52.3%)	99 (58.9%)	0.146
SCD in first-degree relative (n = 1,393)	146 (10.3%)	88 (9.3%)	58 (12.5%)	0.159	78 (9.1%)	45 (8.0%)	33 (11.0%)	0.330	68 (12.3%)	43 11.2%)	25 (15.0%)	0.164
Unexplained syncope (n = 1,432)	125 (8.7%)	80 (8.3%)	45 (9.6%)	0.439	55 (6.3%)	34 (5.9%)	21 (7.0%)	0.546	70 (12.6%)	46 (11.9%)	24 (14.2%)	0.456
NYHA functional class ≥2 (n = 1,416)	340 (24.0%)	213 (22.4%)	127 (27.3%)	0.042	201 (23.2%)	127 (22.4%)	74 (24.8%)	0.428	139 (25.3%)	86 (22.5%)	53 (31.9%)	0.019
NSVT (n = 1,214)	78 (6.2%)	53 (6.2)	25 (6.1%)	0.594	36 (4.1%)	23 (4.0%)	13 (4.3%)		42 (7.6%)	30 (7.8%)	12 (7.1%)	
Beta-blockers	549 (38.3%)	191 (40.6%)	358 (37.2%)	0.222	332 (37.9%)	214 (37.2%)	118 (39.1%)	0.5990	217 (39.0%)	144 (37.2%)	73 (43.2%)	0.183
Phenotype												
LVMWT (mm) (n = 1,386)	17 ± 7.2	17.1 ± 7.23	16.6 ± 7.1	0.2317	15.4 ± 6.6	15.7 ± 6.7	14.8 ± 6.4	0.081	19.4 ± 7.4	19.3 ± 7.5	19.8 ± 7.3	0.416
LVMWT *z*-score (n = 1,228)	11.1 ± 6.9	11.1 ± 7.1	11.1 ± 6.5	0.9006	11.1 ± 7.0	11.3 ± 7.4	10.6 ± 6.3	0.187	11.1 ± 6.8	10.8 ± 6.8	11.8 ± 6.6	0.129
LVEDD (mm) (n = 1,186)	37.1 ± 8.1	37.7 ± 8.2	36.0 ± 7.9	0.001	33.8 ± 7.5	34.0 ± 7.5	33.4 ± 7.4	0.275	42.1 ± 6.3	42.8 ± 6.1	40.5 ± 6.7	0.0003
LVEDD *z*-score (n = 1,072)	−1 0.4 ± 1.7	−1.3 ± 1.7	−1.4 ± 1.7	0.4319	−1.2 ± 1.7	−1.2 ± 1.8	−1.2 ± 1.6	0.940	−1.6 ± 1.6	−1.5 ± 1.5	−1.8 ± 1.7	0.113
LA diameter (n = 1,070)	31.5 ± 9.0	31.7 ± 8.7	31.2 ± 9.6	0.412	28.5 ± 8.5	28.7 ± 8.3	28.3 ± 8.9	0.585	35.6 ± 8.1	35.4 ± 7.8	36.2 ± 8.6	0.310
LA diameter *z*-score (n = 1,022)	1.5 ± 2.5	1.4 ± 2.4	1.8 ± 2.7	0.0083	1.4 ± 2.6	1.3 ± 2.5	1.6 ± 2.8	0.162	1.6 ± 2.4	1.4 ± 2.3	2.1 ± 2.5	0.0056
Maximal LVOT gradient (n = 1,266)	8 (5, 17)	8 (5, 19)	7.5 (5, 16)	0.4801	8 (5, 24)	9 (5, 28)	8 (5, 16)	0.1403	7 (5, 12)	7 (5, 12)	7 (5, 16)	0.714
LVOT ≥30 mm Hg (n = 1,266)	242 (19.1)	164 (19.4)	78 (18.6)	0.751	172 (22.8%)	119 (24.3%)	53 (20.1%)	0.184	70 (13.7%)	45 (12.6%)	25 (16.1%)	0.281
LVOTO ≥50 mm Hg (n = 1,266)	177 (14.0)	126 (14.9)	51 (12.2)	0.192	129 (17.1%)	93 (19.0%)	36 (13.6%)	0.061	48 (9.4%)	33 (9.2%)	15 (9.7%)	0.870

Values are median (IQR), n (%), or mean ± SD.

HCM = hypertrophic cardiomyopathy; ICD = implantable cardiac defibrillator; LA = left atrial; LVEDD = left ventricular end diastolic diameter; LVMWT = left ventricular maximal wall thickness; LVOT = left ventricular outflow tract; LVOTO = left ventricular outflow tract obstruction; NSVT = non-sustained ventricular tachycardia; NYHA = New York Heart Association; SCD = sudden cardiac death; VF = ventricular fibrillation; VT = ventricular tachycardia.

**Central Illustration:**
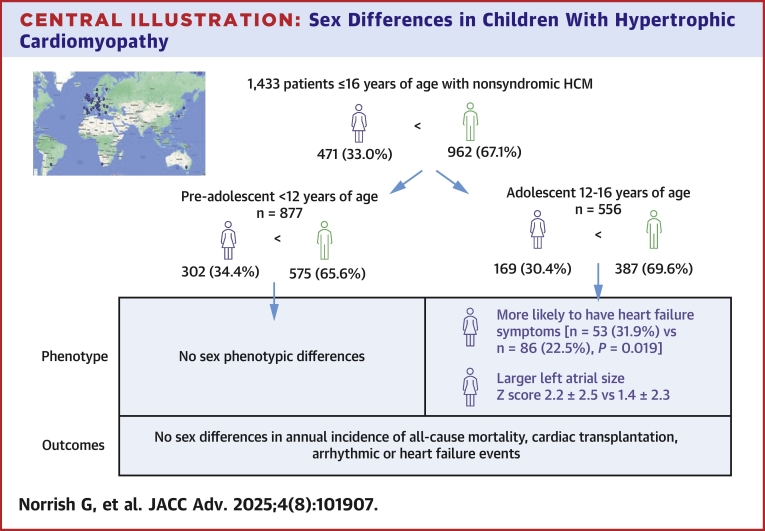
Sex Differences in Children With Hypertrophic Cardiomyopathy HCM = hypertrophic cardiomyopathy.

### Baseline clinical phenotype

The baseline clinical characteristics are described in Table 1. Female patients were more likely to have heart failure symptoms at presentation (male n = 213, 22.4% vs female n = 127, 27.3%; *P* = 0.042) but did not otherwise differ in symptomatology or use of cardiac medications. A family history of HCM was seen in half of the cohort (n = 758, 53.4%), with no difference between males and females. Genetic testing was performed in 917 patients (64.0% of the cohort). There was no sex difference in the proportion of individuals who underwent genetic testing (male n = 609, 63.3% vs female n = 308, 65.4%) or in whom a sarcomeric variant was identified (male, n = 414, 68.0% vs female, n = 213, 69.2%; *P* = 0.1307). Variants in MYH7 were the most frequently reported for both sexes (male, n = 171, 41.5%) vs female, n = 94, 44.1%) but variants in thin filament proteins were more frequently reported in female patients (25.4% vs 19.1%; *P* = 0.048).

The BSA-corrected MLVWT did not differ by sex and there was a similar proportion of male and female patients with resting LVOTO (male n = 164, 19.4% vs female n = 78, 18.6%; *P* value 0.751). Female patients had a higher mean BSA-corrected (*z*-score) LA diameter (male 1.4 ± 2.3 vs female 1.8 ± 2.7; *P* = 0.008).

### Age-related differences in phenotype

The clinical phenotype of patients presenting in preadolescence (≤12 years) and adolescence (>12 years) was compared (Table 1, central illustration). No sex differences in baseline clinical characteristics of phenotype were seen for patients presenting in preadolescence. Adolescent female patients were more likely to have heart failure symptoms at presentation (male, n = 86, 22.5% vs female, n = 53, 31.9%; *P* = 0.019) and a dilated left atrium (LA diameter *z*-score male 1.4 ± 2.4 vs female 2.1 ± 2.5; *P* = 0.0056).

### Clinical follow-up

Patients were followed up for a median of 5.3 years (IQR: 2.9, 8.0); follow-up was censored at the age of 22 years for 283 (19.7%) patients. Female patients had a longer median follow-up (male 5.0 [IQR: 2.7, 8.0] vs female 6.0 [IQR: 3.4, 8.5]; *P* = 0.0028) but were a similar age at last review (male 17.1 [IQR: 13.1, 20.6] vs female 17.1 [IQR: 13.3, 20.8]; *P* = 0.5733). Over this time, 141 (9.8%) patients underwent a myectomy (preadolescent n = 100 [12.9%], adolescent n = 41 [7.3%]) and 397 (27.8%) had an ICD implanted for primary (n = 335, 84.6%) or secondary (n = 59, 14.9%) prevention, with no significant differences between male and female patients (Table 2). Female patients were more likely to have a pacemaker implanted (female n = 17, 3.6% vs male 13. 1.4%; *P* = 0.005) but there were no sex differences in the indication for pacemaker.

**Table 2:** Interventions and Outcomes by Sex

	Whole Cohort (N = 1,433)	Male (n = 962)	Female (n = 471)	*P* Value
Myectomy	141 (9.8)	93 (9.7)	48 (10.2)	0.0978
ICD	397 (27.7%)	253 (26.3%)	144 (30.6%)	0.089
Indication for ICD				
Primary prevention	335 (84.6%)	209 (82.6$)	126 (88.1%)	0.244
Secondary prevention	59 (14.9%)	42 (16.6%)	17 (11.9%)	
Unknown	2 (0.51%)	2 (0.79%)	0	
ICD therapy	91 (20.3)	62 (21.5)	29 (18.0%)	0.834
ATP	4	3	1	
Appropriate shock	55	38	17	
Inappropriate shock	11	9	2	
Pacemaker	30 (2.1%)	13 (1.4%)	17 (3.6%)	0.005
Indication for pacemaker				
Sinoatrial disease	5	3	2	0.628
AV node disease	13	6	7	
LVOT obstruction	9	3	6	
Unknown	1	0	1	
Heart transplant	36 (2.5%)	22 (2.3%)	14 (3.0%)	0.436
Death	79 (5.5%)	54 (5.6%)	25 (5.3%)	0.811
Cause of death				
Sudden death	53	47	16	0.334
Heart failure related	11	6	5	
Stroke	0	0	0	
Other CV	3	1	2	
Non-CV	4	2	2	
Unknown	8	5	3	
Heart failure endpoint, n (%)	48 (3.5%)	28 (2.9%)	20 (4.3%)	0.187
Incidence heart failure endpoint per patient year	0.56 (0.42-0.74)	0.51 (0.35-0.73)	0.67 (0.43-1.03)	0.342
Age at heart failure endpoint	17 (13, 20,5)	17 (12.8, 20.3)	17.1 (13.2, 20.8)	0.515
MACE, n (%)	145 (10.1%)	102 (10.6%)	42 (8.9%)	0.319
Incidence MACE per patient year	1.72 (1.46-2.02)	1.88 (1.55-2.29)	1.41 (1.04-1.91)	0.112
Age at MACE endpoint	16.9 (12.9, 20.3)	16.9 (12.5, 20.1)	16.9 (1.2, 20.7)	0.466
All-cause mortality/Tx	114 (8.0)	79 (7.9%)	38 (8.1%)	0.912
Age at time mortality/tx endpoint	14.5 (11.5, 17.1)	14.6 (11.5, 16.9)	14.3 (11.5, 17.3)	0.487
Incidence all-cause mortality/tx per patient year	1.3 (1.09-1.58)	1.34 (1.07-1.68)	1.26 (0.92-1.74)	0.728

ATP = anti-tachycardia pacing; AV = atrioventricular; CV = cardiovascular; ICD = implantable cardiac defibrillator; MACE = major arrhythmic cardiac event; other abbreviation as in Table 1.

### Outcomes

**Figure 2:**
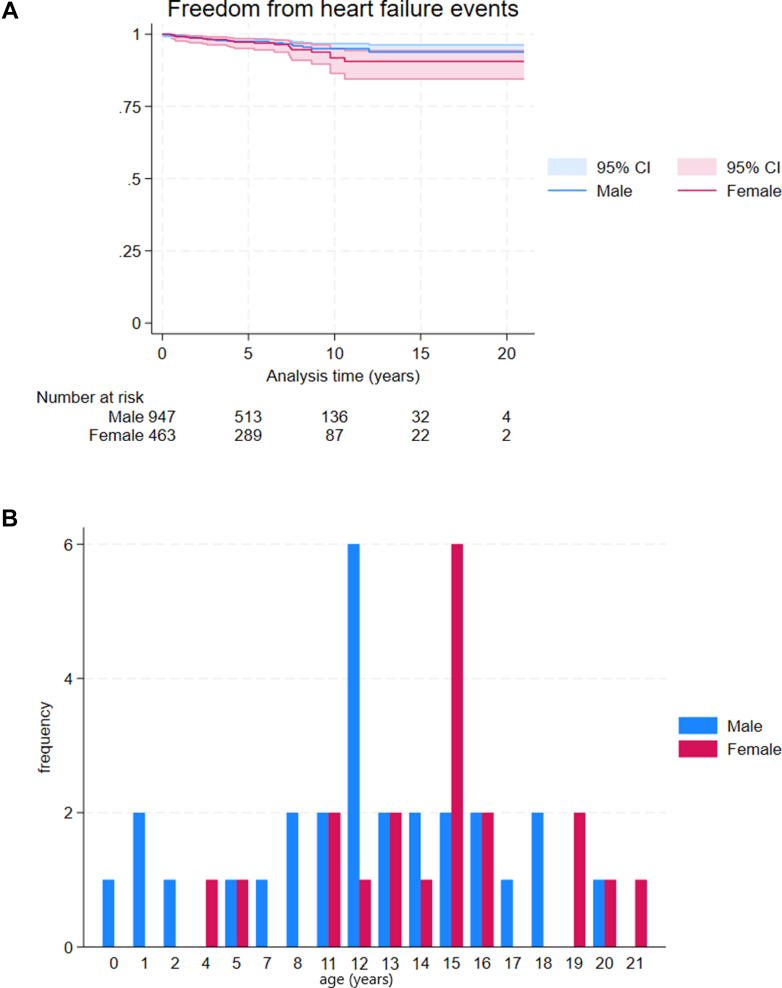
Heart Failure Events by Sex (A) Graph shows cumulative incidence of heart failure events during follow-up by sex (male 0.51 per 100 patient-years [95% CI: 0.35-0.73] vs female 0.67 per 100 patient-years [95% CI: 0.43-1.03]; *P* = 0.342) (B) Bar chart shows age at the time of heart failure event by sex.

**Figure 3:**
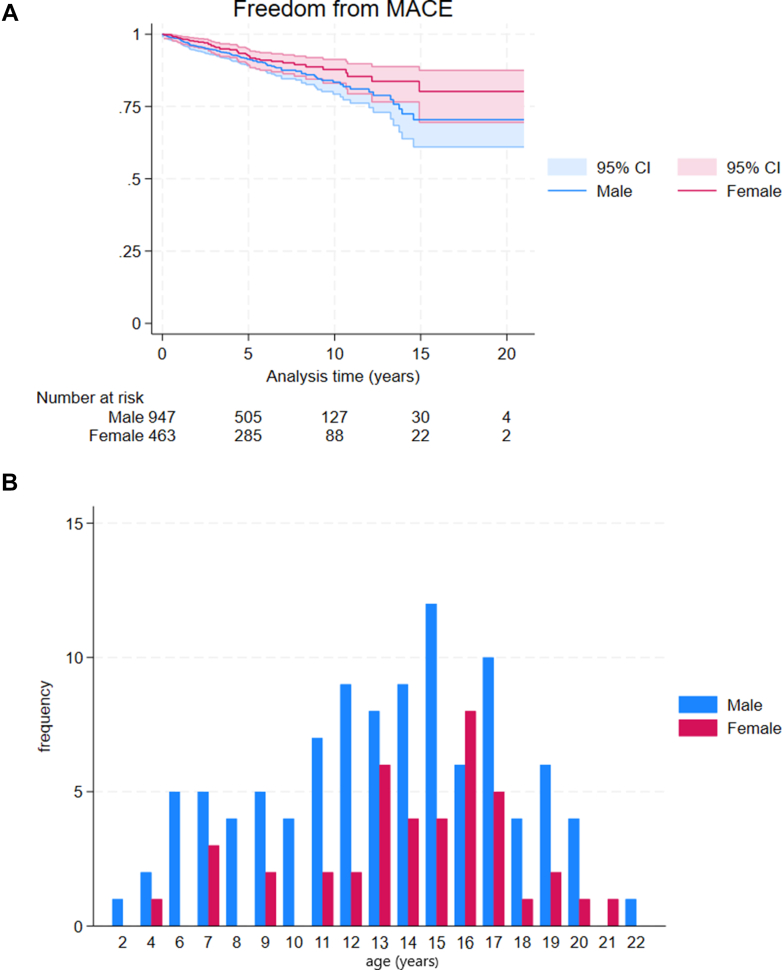
Arrhythmic Events by Sex (A) Graph shows freedom from arrhythmic events during follow-up by sex (male 1.88 per 100 patient-years [95% CI: 1.55-2.29] vs female 1.41 per 100 patient-years [95% CI: 1.04-1.91]; *P* = 0.112) (B) Bar chart shows age at the time of arrhythmic event by sex. MACE = major arrhythmic cardiac event.

Seventy-nine (5.5%) patients died during follow-up (sudden cardiac death n = 53, heart failure related n = 11, other CV, n = 3, non-CV n = 4, unknown n = 8) and 36 (2.5%) underwent cardiac transplantation. The annual incidence of all-cause mortality or transplant did not differ by sex (male 1.34 per 100 patient years [95% CI: 1.07-1.68] vs female 1.26 per 100 patient years [95% CI: 0.92-1.74]). Heart failure events occurred in 48 patients (3.5%); the annual incidence (male 0.51 per 100 patient-years [95% CI: 0.35-0.73] vs female 0.67 per 100 patient-years [95% CI: 0.43-1.03], *P* = 0.342) and age at the time of heart failure events did not differ between male and female patients (Figure 2). One hundred and forty-five patients (10.1%) experienced one or more MACE over follow-up. The annual incidence of MACE did not differ between female and male patients (male 1.88 per 100 patient-years [95% CI: 1.55-2.29] vs female 1.41 per 100 patient-years [95% CI: 1.04-1.91]; *P* = 0.112) and arrhythmic events occurred at a comparable age (Figure 3). Adjusting for age at diagnosis did not affect the time-to-event results (Supplemental Table 1).

## Discussion

To our knowledge, this study is the first systematic description of sex differences in childhood-onset HCM. No sex differences were seen in preadolescent patients, but adolescent females are more likely to experience heart failure symptoms and have evidence of impaired diastolic function at baseline. A difference in the phenotype of preadolescent and adolescent patients suggests that sex hormones could be an important modifier for phenotype during childhood. Despite these phenotypic differences, outcomes are similar during childhood and adolescence.

### Phenotypic sex differences

In adult cohorts, clear phenotypic differences have been described between male and female patients and are associated with differences in outcomes. Women have a higher prevalence of LVOTO and diastolic impairment at presentation.^2^^,^^6^^,^^7^ A smaller left ventricular cavity size has been proposed to underlie these phenotypic differences,^21^ which could explain why females are more likely to report heart failure symptoms and reduced exercise capacity independent of the presence or absence of obstruction. Men tend to have more hypertrophy, as measured by MLVWT, but data from the ShARE registry suggest that when measurements are corrected for BSA this association is reversed, with females having higher BSA-corrected MLVWT measurements.^2^^,^^3^^,^^6^^,^^7^ The explanations for these differences have been attributed, at least partly, to a later diagnosis in women who are more likely to present at an older age with symptoms of heart failure and possibly more advanced disease. In contrast to adult cohorts, in the present study, there was no sex difference in the proportion of patients with obstructive disease at baseline or the extent of hypertrophy. Despite this, adolescent female patients were more likely to have a dilated left atrium, an echocardiographic surrogate marker for diastolic dysfunction, and heart failure symptoms at presentation. The presence of such phenotypic differences in childhood suggests that the observed differences seen in adult patients cannot be explained solely by late presentation. The difference in phenotype observed between the preadolescent and adolescent female patients could suggest that sex hormones may play an important role. Recent studies have shown differences in the protein expression of myectomy samples from male and female patients,^22^ suggesting underlying pathophysiological differences may exist between the sexes that could also contribute to the observed differences in phenotype.

### Sex differences in the genetics of childhood HCM

Sarcomeric HCM is inherited as an autosomal dominant trait and so would be predicted to have an equal sex prevalence. However, studies have repeatedly described a male preponderance making up around two-thirds of published adult cohorts.^3^, ^4^, ^5^, ^6^ This disparity remains unexplained, but proposed mechanisms include a protective effect of sex hormones reducing the penetrance of sarcomeric variants^4^^,^^11^^,^^23^; higher incidence of nongenetic risk factors associated with disease in male patients (eg, hypertension, type II diabetes^6^^,^^24^^,^^25^); and diagnostic bias, with females being less likely to be diagnosed through screening and more likely to present with symptoms that may be misinterpreted by clinicians.^7^ In this study, we have shown that the male predominance described in adulthood extends into childhood and is present even in infancy. This suggests that the underlying mechanism is complex but unlikely to be solely driven by sex hormone expression or the presence of other CV risk factors that are typically absent in this young population. Reason for diagnosis was not collected in this study so we are unable to speculate on sex differences in screening, but a similar proportion of male and female patients had a family history of HCM.

Adult studies have described sex differences in the yield of genetic testing, with sarcomeric variants more likely to be detected in female patients.^2^^,^^10^^,^^26^ In agreement with previous studies, the yield of genetic testing was higher in our childhood cohort compared to comparable adult populations,^14^^,^^27^ but importantly, the yield of genetic testing was similar for male and female patients. This may reflect the absence of the “typical” gene-elusive adult individual who is more likely to be male, have coexisting traditional CV risk factors and likely polygenic inheritance pattern.^9^^,^^28^ Although the yield of genetic testing did not differ by sex, the genes affected varied, with thin filament protein variants more commonly reported in female patients. It is beyond the scope of this paper to investigate sex differences in the expression of sarcomeric variants but these data raise the possibility that the modifier effect of sex may differ for different sarcomere protein genes.^29^

### Sex differences in outcomes and management

In adults, women have been shown to have a 50% higher excess mortality compared with male patients, which is largely attributed to heart failure–related deaths.^2^^,^^10^ In contrast, in this study, no sex differences were seen in the incidence of all-cause mortality, heart failure or arrhythmic events occurring during childhood or early adulthood. This may be explained by the absence of longer term follow-up into adulthood; data from SHaRe have previously demonstrated that events in childhood are predominantly arrhythmic in nature but an increasing prevalence of heart failure events is seen in those diagnosed in childhood during follow-up into adulthood.^14^ It is possible that a sex difference may have been seen if surrogate markers of heart failure death including heart failure admissions, impaired systolic function, or B-type natriuretic peptide levels had been investigated.

In this study, there was a trend toward a higher rate of ICD implantation (predominantly primary prevention devices) in female patients, but this did not reach statistical significance. This may be related to the higher median LA diameter in female patients, given that this is one of 5 clinical variables known to predict the risk of sudden death events in childhood.^20^^,^^30^ However, it may also reflect a clinician-perceived difference in risk between male and female patients, or possible sex differences in the threshold of what is an acceptable risk for patients and their families themselves. Future studies to assess sex differences in perception of risk in HCM are warranted. Our findings are similar to previous reports from adult cohorts and suggest that, once patients are diagnosed, there is sex equity in terms of access to specialist care and interventions.^7^

### Study Limitations

This study has inherent limitations due to its multicenter and retrospective design, including missing data and incomplete recruitment of eligible patients. Variations in clinical assessment and patient management are inevitable as patients were recruited from multiple centers and different geographical locations. Assessing symptom burden in this young population can be challenging for a multitude of nonclinical reasons, including under-reporting, poor recall and intentional, or nonintentional, limitation of activities due to the diagnosis. Clinician-determined symptom assessment tools such as NYHA and Ross heart failure classifications are therefore inherently subjective and susceptible to bias. This study did not collect information on serum biomarkers (eg, brain natriuretic peptide) or cardiopulmonary exercise testing that could provide more objective measures of a patient’s cardiopulmonary fitness. Future studies that incorporate such variables could provide interesting insights into symptoms in childhood disease. It is beyond the scope of this study to describe sex differences in disease progression as serial data were not available for all patients. Genetic testing was performed on a clinical basis with significant variability in testing strategy at collaborating centers. It is therefore beyond the scope of this paper to investigate sex differences in the yield of genetic testing.

## Conclusions

This study has shown sex differences in pediatric-onset HCM. Adolescent female patients are more likely to have features of diastolic impairment and experience heart failure symptoms. Despite differences in phenotype, outcomes during childhood and young adulthood are not different. A difference in the phenotype of preadolescent and adolescent patients suggests that sex hormones could be an important modifier for phenotype during childhood. Further studies are required to explore the underlying mechanisms for this observed difference.

Perspectives **COMPETENCY IN PATIENT CARE:** Childhood-onset HCM is most commonly caused by sarcomeric mutations inherited as an autosomal dominant trait, but clinical disease expression is reported more commonly in males. However, although there were no differences in clinical outcomes, female patients were more likely to experience heart failure symptoms and have evidence of impaired left ventricular diastolic function.

**TRANSLATIONAL OUTLOOK:** A difference in the phenotype of preadolescent and adolescent patients suggests that sex hormones could be an important modifier for phenotype during childhood. Further studies are required to explore the underlying mechanisms for this observed difference.

## Funding support and author disclosures

Dr Norrish is supported by Great Ormond Street Hospital Children’s Charity. Drs Field and Kaski are supported by Max’s Foundation and Great Ormond Street Hospital Children’s Charity. Dr Kaski is supported by a Medical Research Council Clinical (MRC)-National Institute for Health Research (NIHR) Clinical Academic Research Partnership (CARP) award. The work reported in this publication was also funded by the Italian Ministry of Health, RC-2024-2789983 (EB). All other authors have reported that they have no relationships relevant to the contents of this paper to disclose.
