# Supplementary material for: Sex Differences in Children and Adolescents With Hypertrophic Cardiomyopathy
Source: JACC Adv. 2025 Jul 5;4(8):101907. doi: 10.1016/j.jacadv.2025.101907 (PMC12272437; doi:10.1016/j.jacadv.2025.101907)
Supplement: Supplemental Material [file mmc1.docx]

|  |  | Whole cohort (n=1433) | Male (n=962) | Female (n=471) | P value |
| --- | --- | --- | --- | --- | --- |
| Adjusted for age at diagnosis | Incidence heart failure end point per patient year | 0.56 (0.42-0.74) | 0.51 (0.35-0.73) | 0.67 (0.43-1.03) | 0.319 |
|  | Incidence MACE per patient year | 1.72 (1.46-2.02) | 1.88 (1.55-2.29) | 1.41 (1.04-1.91) | 0.1731 |

Supplemental Table 1: Time to event analysis by sex adjusted for age at diagnosis
